# Multilevel Causal Analysis of Socio-Psychological and Behavioral Factors of Health Providers and Clients That Affect Health Behavioral Modification in Obesity

**DOI:** 10.5539/gjhs.v7n6p117

**Published:** 2015-04-03

**Authors:** Ungsinun Intarakamhang, Patrawut Intarakamhang

**Affiliations:** 1 Behavioral Science Research Institute, Srinakharinwirot University, Bangkok, Thailand; 2 Department of Physical Medicine and Rehabilitation, Phramongkutklao College of medicine and Hospital, Bangkok, Thailand

**Keywords:** health behavioral modification, multilevel, obesity, self-care, self-efficacy, self-regulation

## Abstract

The Comprehensive Lifestyle Intervention, which integrates behavioral therapy, is the main ideal management of the clients with obesity. Various socio-psychological factors can affect outcome of the program.

**The Purposes:**

To determine the socio-psychological factors at the client and provider groups that affect health behavior modification (HBM) in obese clients, and to investigate the cross-level interaction of factors that affect HBM. The samples included 87 health providers and 412 clients using stratified random sampling. Hierarchical Linear Model was used to analyze in a questionnaire with reliability of 0.8-0.9.

**Results:**

1) for the clients: 1.1) Attitudes towards healthy behavior (AHB), health-related knowledge, and trust in the provider predicted self-efficacy at 49.40%; 1.2) AHB and support from the provider predicted self-regulation at 75.50%; and 1.3) AHB, trust in the provider and support from the provider predicted self-care at 26.6%. 2) for the health providers: 2.1) Health quotient (HQ), project management (PM), support from the team, and the team emotional quotient (EQ) predicted self-efficacy at 71.30%; 2.2) PM and HQ predicted self-regulation at 51.60%; and 2.3) PM, team EQ and HQ predicted self-care at 77.30%., 3) No cross-level interaction of factors between the clients and the providers was identified to affect HBM.

**Conclusion:**

The obese client’s AHB is the factor that significantly influenced self-efficacy, self-regulation and self-care (3SELF).At the health provider level, both HQ and PM significantly influenced 3SELF. Behavioral.

## 1. Introduction

Obesity and overweight are becoming the leading causes of morbidity and mortality worldwide due to the consequences of metabolic syndrome, e.g., hypertension, type 2 diabetic mellitus, dyslipidemia, and coronary heart disease. In 2008, the number of overweight adults worldwide was 1.5 billion. In the United States, there were more than 7.8 million obese adults in 2009-2010 (The Obesity Expert Panel, 2013). The National Health Examination Survey indicated that the risk of obesity in Thai individuals increased from 20 to 25% between 1991 and 1996 and from 32 to 37% between 2004 and 2009 (Health Systems Research Institute, 2006, 2010). Thus, approximately one-third of the Thai population, i.e., 20 million individuals, are obese. In the last decade, the incidence of obesity in Thailand has increased by 130.6%.

Because the course of obesity and the related diseases are chronic, the government must pay higher healthcare costs for obese individuals than for normal individuals. In the United States, a strategy to control obesity has been developed; guidelines (2013) for the management of overweight and obesity in adults have been released, and the most important concept is “comprehensive lifestyle modification” (The Obesity Expert Panel, 2013). This concept consists of 3 components, including 1) a reduced-calorie diet, 2) an increase in physical activity to increase the number of calories burned and 3) behavioral therapy (Savage, 2013).

Behavioral therapy is typically included in an obesity management program, as well as in comprehensive cardiac rehabilitation programs. Behavioral therapy supports a structured behavioral change that includes regular self-monitoring of food intake (Savage, 2013), physical activity and weight control. Many concepts of behavioral modification have been accepted theoretically. The social cognitive learning theory (Bandura, 1986) explained that patterns of behaviour are established by interactive learning between individuals and the social environment. Social support theory described the importance of the perception and the actuality that an individual is cared for, has assistance available from other individuals, and is part of a supportive social network. Social support includes the entire range of disciplines of psychology, medicine, sociology, nursing, public health and social work and has been linked to many benefits for both physical and mental health. Studies of many different variables, such as social support (House & Kahn, 1985), services with an emphasis on an individual or client-centred approach (Rogers, 1987), self-care (Orem, 1991), a positive attitude towards healthy behaviours (Bagozzi, 1992), project management (Maylor, 1996), self-efficacy (Bandura, 1997), self-regulation (Schunk & Zimmerman, 1997), HQ (Li Enchang, 2001), the leader’s role (Bruke et al., 2006), the team emotional quotient (Druskat & Wolff, 2001), trust in the service provider (Ingchatcharoen, Boonprakob, & Intarakamhang, 2009) and the variables of the PROMISE model (Intarakamhang, 2009), have emphasised the role of comprehensive behavioral modification in conditions influenced by the prolonged presentation of behaviors related to poor health. Intarakamhang (2012) created a comprehensive health behavior program, including guidelines for healthy behavior, in which the main concept was the 3SELF principle (self-efficacy, self-regulation, self-care) based on PROMISE (P-Positive reinforcement, R-Results-based management, O-Optimism, M-Motivation, I-Individual or Client centeredness and SE-Self-esteem). This program was then implemented in 4,649 individuals at metabolic risk who were involved in 30 projects. The clients had a significantly reduced average BMI, blood pressure and waist circumference after completing the program (p < .05), and the clients in the non-obese group (BMI < 24.5) presented significantly better self-care behaviour compared with the group with a BMI > 24.5 (p < .05).

According to interviews with the project managers, the factors that were thought to contribute most to the success of the project were the project’s policies, the effective teamwork and positive relationships amongst the clients, trust in the medical staff, and the incorporation of interesting activities and rewards. Thus, the question arises as to whether there may be some correlation, linkage or interaction between health provider and/or client variables and the 3SELF principles that determines the success of health behavioral modification (HBM) in clients.

## 2. Objective

**Figure 1:**
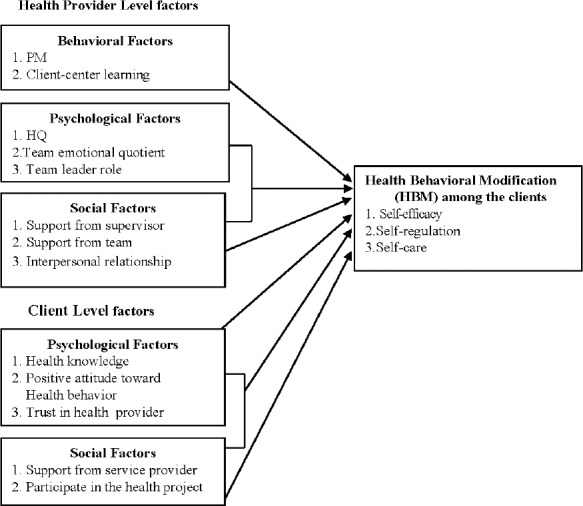
The theoretical framework of multilevel causal analysis of socio-psychological and behavior factors affecting HBM of the clients
2) To investigate the cross-level interaction of factors between the client and health provider groups that influence the self-efficacy of health care utilization, self-regulation and self-care for obese clients (Figure 1).

1) To determine the socio-psychological factors that influence the client and provider group levels that affect HBM in obese clients, such as self-efficacy of health care, self-regulation and self-care behavior (Figure 1).

## 3. Method

### 3.1 Sample

The sample size was determined based on the size required to demonstrate a consistent pattern of causal relationships at multiple levels. The group sizes were based on the concept of Snijders and Bosker (1999), who suggested that an adequate sample size for a group should be at least 20 and that to study the causal relationships between individuals, the sample size at the client level should not be less than 300. Thus, this research included 20 groups at the project level for a total of 87 health providers, with 4-5 providers from each project, and 412 clients selected by proportion-stratified random sampling. The study used “project” as the stratification variable and determined the ratio of projects: participants at 1:20.

### 3.2 Inclusion Criteria

The clients were selected from individuals who were at risk of obesity (BMI>23) according to the classification of obesity (Table 1).

**Table 1:** Classification of overweight and obesity (Savage, 2013)

Weight class	BMI(Kg/m^2^)
Normal	18.5-24.9
Overweight	25.0-29.9
Class 1 obesity	30.0-34.9
Class 2 obesity	35.0-39.9
Class 3 obesity	≥40

### 3.3 Setting

For the projects related to risk behaviors, during the years 2010-2012, hospitals that were funded by the National Health Security Office of Thailand trained the project leaders and teams in technical knowledge related to the 3SELF behaviors and the principles of PM based on the PROMISE Model of Intarakamhang (2009). These training programs provided guidance for the successful implementation of similar programs that target diseases with behavior-based risk factors similar to obesity, providing a way for clients to participate in a health modification project wherever it is convenient for them to access a program. The health providers were selected at the project level and included project managers and service providers engaged in behavioral modification programs for individuals at risk of obesity, both in public and private hospitals that received funding from the National Health Security Office of Thailand, for a total of 150 projects. The clients participated in an HBM program conducted by the hospitals during the year 2012, which included a total of 25,000 clients.

### 3.4 Measurements

The research instruments included two questionnaires to measure 15 variables. The first instrument was a 112-item questionnaire with a 6-point Likert scale for the obese clients to assess the influence of socio-psychological factors and self-efficacy, self-regulation and self-care on the outcome of the HBM program. The responses were rated from 1 to 6, corresponding to “not at all” to “definitely true”. The second instrument was a 99-item questionnaire with a 6-point Likert scale for the health providers to assess the influence of socio-psychological factors on the outcome of the HBM program. The responses were rated from 1 to 6, corresponding to “minimum” to “definitely maximum”. All 14 variables passed the content validity by 3 qualified individuals and a trial that included 50 individuals with the reliability of Cronbach’s alpha coefficient between .810 and .975. The data were collected from March – July 2013.

### 3.5 Statistical Analysis

Hierarchical Linear Model (HLM).

## 4. Results

The groups of clients included 412 individuals who weighed more than 80 kg (27.40%) and had a height less than 155.00 cm (29.60%). The majority of the individuals (52.67%) were classified as obese at level 1, 29.37% were classified as obese at level 2, and 17.96% were classified as overweight.

The coefficients of the matrix decision variable factors between the dependent variables in the client group ranged from 0.139 to 0.776 (p < .01), and the relationship between the matrix variable factors and the dependent variables in the service provider group ranged from 0.019 to 0.847. Thus, the result does not indicate multicolinearity because Kline (2005) indicated that the correlation coefficient between variables should not be higher than 0.85.

Research based on hypothesis 1 tested the influence of psychosocial factors in the client group on the health-related behavior of obese clients, self-efficacy, self-regulation and self-care as follows (Table 2).

**Table 2:** Factors that influence, at the client level, self-efficacy, self- regulation and self-care of clients with obesity

Fixed Effects	Self-efficacy		Self-regulation		Self-care behavior	

	Coefficient(β)	p-value	Coefficient(β)	p-value	Coefficient(β)	p-value
Intercept	4.269	**.000**	4.321	**.000**	3.953	**.000**
Psychological factor						
-good attitude toward healthy behavior	.487*	**.000**	.656*	**.000**	.401*	**.000**
- Health knowledge	.074*	**.002**	.067	**.127**	.018	**.663**
- Trust in health provider	.181*	**.009**	.096	**.322**	.160*	**.017**
Social factor						
- Support from providers	.062	**.264**	.138*	**.049**	.123*	**.023**
-Participate in the health project	.051	**.395**	.075	**.377**	.119	**.055**

Random Effects	S.D.	χ^2^	S.D.	χ^2^	S.D.	χ^2^

-Random variation at health provider level	**.148**	61.177*	**.071**	28.058	**.109**	35.436*
- Random variation at the client level	**.427**		.471		**.503**	
R^2^ at the client level	.494		.755		.266	

-Psychosocial factors, including a positive attitude towards healthy behavior, knowledge about health and trust in the health providers, influenced the self-efficacy of the clients with coefficients (β) of 0.487, 0.274 and 0.181, respectively, and significance (t = 8.060, t = 3.206 and t = 2.639, respectively, P < .05); these findings indicated that collectively, the factors could explain 49.40% of the variance in self-efficacy among the obese clients.

-Psychosocial factors, including a positive attitude towards healthy behavior and support received from a health provider influenced the self-regulation of clients with obesity with coefficients of 0.656 and 0.138, respectively (t = 10.382 and t = 1.969, respectively, P < .05); these two factors combined explained 75.50% of the variance in the self-directed behavior of the clients.

-Psychosocial factors, including a positive attitude towards healthy behavior, trust in the health providers and support from the providers, influenced the self-care of clients with obesity with coefficients of 0.401, 0.160 and 0.123, respectively (t = 6.562, t = 2.402 and t = 2.288, respectively, P < .05); these factors combined explained 26.6% of the variance in self-care.

Psychosocial and health project management factors of the health providers that influenced clients with obesity in accordance with hypothesis 2 (Table 3).

**Table 3:** Results of testing the influence of psychosocial and behavioral factors amongst providers, based on self-efficacy, self-regulation, and self-care on the behavior of patients with obesity

Fixed Effects	Self-efficacy		Self-regulation		Self-care behavior	

	Coefficient(β)	p-value	Coefficient(β)	p-value	Coefficient(β)	p-value
(Intercept)	.428	**.000**	4.330	**.000**	3.970	**.000**
**Psychological Factors**						
- HQ	.713*	**.015**	.531*	**.047**	.499*	**.029**
- Team emotional quotient	.567*	**.049**	.428	**.328**	.576*	**.047**
- Team leader role	**.070**	**.714**	.108	**.596**	.051	**.760**
**Social factors**						
-Support from supervisors	**. 212**	**.157**	.040	**.730**	.003	**.973**
-Support from team	.603*	**.045**	.222	**.406**	.076	.214
-Interpersonal relationship	**.325**	**.359**	-.228	**.585**	.438	**.271**
**Behavioral factors**						
- PM	.660*	**.040**	.659*	**.040**	.709*	**.006**
- Client center learning management	**.387**	**.396**	.045	**.905**	-.183	**.602**

**Random Effects**	**S.D.**	**χ^2^**	**S.D.**	**χ^2^**	**S.D.**	**χ^2^**

- Random variation factor at health provider	**.292**	64.626*	**.278**	46.562*	**.241**	52.278*
- Random variation at client level	.599		**.701**		**.587**	
R^2^ at the health provider level	769		.516		.773	

-Psychosocial and behavioral factors of the health providers that influenced the self-efficacy of the clients with regard to health care, including HQ, PM, support from colleagues and the team emotional quotient, presented influence coefficients of 0.713, 0.660, 0.603 and 0.567, respectively, with significance at .05 (t = 2.910, t = 2.336, t = -2.263 and t = 2.247, respectively). These combined factors related to the providers explained 76.90% of the variance in the self-efficacy of the obese clients.

-Psychosocial and behavioral factors of the health providers that influenced the clients’ self-regulation, such as PM and HQ, presented coefficients of 0.659 and 0.531, respectively, and a significance of .05 (t = 2.493 and t = 2.233, respectively); these factors combined explained 51.60% of the variance in the self-regulation behavior of the obese clients.

-Psychosocial and behavioral factors of the health providers that influenced the clients’ self-care, such as PM, team emotional quotient and HQ, presented influence coefficients of 0.709, 0.576 and 0.499, respectively, and significance of .05 (t = 2.902, 2.561 and t = 2.521, respectively); these factors combined explained 77.30% of the variance in the self-care behavior of the obese clients.

There was no resolution of the cross-level interaction effect between the psychological and social factors of the health providers and clients that influenced the health behavior modifications of the clients with obesity (Table 4).

**Table 4:** Shows the analyses of the cross-level interaction of variants in the health provider group that influence the client level of self-efficacy, self-regulation and self-care behavior

Fixed Effects	Self-efficacy		Self-regulation		Self-care behavior	

	t-ratio	p-value	t -ratio	p-value	t –ratio	p-value
-HQ * Support from providers	**1.722**	**.113**	**.269**	**.793**	**.499**	**.627**
-Team emotional quotient * Participate in the health project	**0.332**	**.746**	**.110**	**.915**	**1.318**	**.214**
-Team leader role* Participate in the health project	**.241**	**.814**	**.716**	**.489**	**.563**	**.584**
-Support from supervisors *positive attitude toward healthy behavior	**.674**	**.514**	**.720**	**.486**	**1.248**	**.238**
-Support from team *Health knowledge	**0.200**	**.845**	**.552**	**.591**	**1.259**	**.234**
-Interpersonal relationship * Trust in health provider	**.706**	**.495**	**.168**	**.870**	**1.073**	**.307**
-PM* Trust in health provider	**.176**	**.864**	**.161**	**.875**	**.430**	**.675**
-Client center*good attitude toward healthy behavior	**.994**	**.342**	**.111**	**.914**	**.134**	**.896**

**Figure 2:**
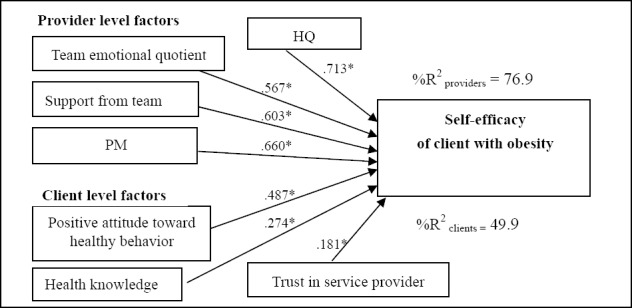
Model of multilevel causal analysis of socio-psychological and behavioral for health provider and the client to self-efficacy of client with obesity

**Figure 3:**
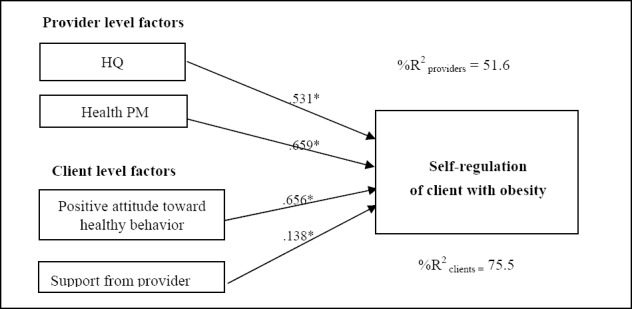
Model of multilevel causal analysis of socio-psychological and behavioral for health provider and the client to self-regulation of client with obesity

**Figure 4:**
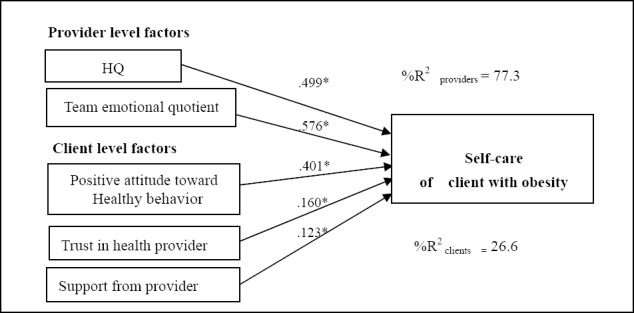
Model multilevel causal analysis of socio-psychological and behavioral for health provider and the client to self-care of client with obesity

There was no evidence of a cross-level interaction of psychosocial factors between the health providers and the clients with obesity. The results are summarized as illustrated in Figures 2-4.

## 5. Discussion

This study demonstrated that the psychosocial characteristics of obese clients influence the outcome of HBM in these patients. In addition, the variable factors at the client level, such as positive attitudes towards health behavior, knowledge about health, trust in the health providers and support from the health providers, had strong influences on self-efficacy, self-regulation and self-care (Table 5). These findings support the social cognitive theory of Bandura (1986) in which human is not simply a result of reinforcement and punishment from the outside; rather, individuals can learn and be taught to control their thoughts, feelings and actions through self-regulation (Zimmerman, 1998). Bandura (1997) stated that when individuals had direct knowledge and experience, their self-efficacy increased. This suggestion is supported by the study of Grey et al. (2004, pp. 10-15), which tested the effectiveness of programs that prevent type 2 diabetes in a group of 44 young individuals, including 22 individuals in the experimental group and 19 individuals in the control group, over a period of 12 months. This program taught nutrition skills and engaged the students in physical activity after school to improve their general level of fitness. These activities led to more significant decreases in HbA1c, weight and body mass index in the experimental group compared with the control group.

**Table 5:** Psycho-social factors of clients and health providers that significantly influenced 3SELF

Psycho-social factors	Client		

	Self-efficacy	Self-regulation	Self-care
**Client level**			
-attitude toward health behavior	Sig.*	Sig.*	Sig.*
-trust in health provider	Sig.*	None	Sig.*
-support from provider	None	Sig.*	Sig.*
-health knowledge	Sig.*	None	None

**Health provider level**			
-HQ	Sig.*	Sig.*	Sig.*
-PM	Sig.*	Sig.*	Sig.*
-EQ	Sig.*	None	Sig.*
-Support from team	Sig.*	None	None

Sig.* means significantly influence at p<.05

In a study by Chirapolsert (2009), attitudinal factors were affected by knowledge related to obesity and weight control in female students at the undergraduate level. The study demonstrated that the greater the awareness of obesity, the more positive the attitudes of the participants towards weight control and corrected behavior, which were associated with subsequent weight loss. A study by Ajzen and Fishbein (1980) stated that the attitude towards action is a large determinant of the outcome. A positive attitude typically induces the intention to do the thing that follows. In contrast, if an individual has a negative attitude, then the intention to act in one’s best interest may not be present. Duangchan, Yoelao, Macaskill, Intarakamhang, and Suprasonsin (2010) applied the concept of Ajzen and Fishbein to study the causal factors related to healthy that prevent obesity in grade 4 primary school children at a demonstration school in Bangkok. The findings related to the participants’ attitude towards health behavior and the influence of health-related knowledge on healthy behaviors were in accordance with Utsahakit (2007), who studied the roles of HQ and information regarding weight control in the improvement of self-efficacy to control body weight. These findings demonstrated that the enhancement of the perception of self-efficacy in middle-aged women resulted in improved behaviors related to weight control and a decrease in body mass index. Thus, knowledge has a direct effect on self-efficacy.

When considering the variable of client trust in the health provider, which reflects a relationship with the prospect of change, a study by Kumnaunsak (2002) with nurses demonstrated that trust significantly affected the operational results.In this study, the perceived support from providers directly influenced self-directed and affected the clients’ self-care. This finding indicated that if clients receive information, are empowered and are provided with care through a variety of resources from the health provider, the clients subsequently change their health behavior related to self-regulation and self-care in accordance with the ideas of Schaefer, Coyne, and Lazarus (1981). These authors stated that social support is important for sustaining one’s focus on achieving a goal. Suriya (2007) studied the effectiveness of weight loss programs by applying the concept of self-regulation with strong social support from nurses. The receipt of social support for weight loss was significantly associated with a reduction in weight, participation in physical activity, moderation of food intake and mood changes in the participants in the experimental group. Wongchan, Intarakamhang, and Boonprakop (2012) studied the influence of mental health and the social environment on the health and nutrition status of personnel in the Department of Health and found that social support from family, colleagues and supervisors could significantly predict health behaviors at 58.0%.

The findings of the present study are consistent with hypothesis 2, which proposed that psychosocial factors and the of project health administrators influence aspects of the risk profile associated with obese patients’ Psychosocial and all factors at the health provider level indicated that there were 4 factors that significantly influenced the clients’ self-efficacy. These factors included the HQ, the team’s emotional quotient, perceived support from the team and health care administration. These factors combined predicted 76.9% of the variance of self-efficacy. Additionally, there were 2 other factors that affected the health providers’ level of influence on the self-regulation of health behavior in obese clients. These factors were the HQ and health management program, which accounted for 51.6% of the variance of self-regulation. These factors must be taken into account when evaluating changes in the risk-related behavior, self-efficacy in healthcare, self-regulation and self-care of obese clients. It is important for health providers to be aware of their role on the team and to care for and support each other because they act as role models of self-care; furthermore, a skilled project management team can provide a positive direction for health behavioral change. Similarly, in a previous study, Druskat and Wolff (2001) stated that when team members concerned the feelings of others, have positive relationships with each other and a clear understanding of each other’s feelings and needs, problem solving is enhanced and clients are more likely to achieve their goals through changes in their behavior.This aspect is known as emotional wellbeing and is one of the important elements of HQ, Diener (2000, p. 34). “Happiness” is associated with good mental health and emotional attitudes that manifest as intelligence, which can benefit other individuals. The ability of health providers to affect a positive change in the clients’ self-efficacy, self-care and self-regulation requires a successful PM (Anbari, Elias, & Voetsch, 2008).

Finally, the study results did not support hypothesis 3 regarding the cross-level interaction between the psychosocial factors at the health provider and client levels that affected the self-efficacy of obese clients. However, regarding self-regulation, PM was the only factor that significantly influenced health knowledge (t = 2.545, p = .027). This finding indicates that the health providers who presented different management styles achieved different magnitudes of change associated with health-related knowledge, which impacted the clients’ self-regulation with regard to healthy. Based on the results obtained for the clients included in this research, we conclude that it is important that health providers have good behavior because the PM directly affects the clients’ health more than the perception that the health provider is a PM. Otherwise, no cross-level interaction effect was identified. Our findings indicate that the clients ‘changes in health behaviours did not vary according to the project group that the clients were in. Thus, clients who participated in all projects achieved the same outcome.

Based on Figures 2, 3, and 4, the psycho-social factors of clients and health providers that significantly influenced the 3SELF principles (self-efficacy, self-regulation and self-care) are presented in Table 4. The client characteristic that significantly influenced all of the 3SELF principles was “positive attitude towards health behavior”. The second and third most significant factors were “trust in health provider” and “support from provider”, respectively. At the health provider level, both HQ and PM significantly influenced all of the 3SELF principles. The second most significant factor was EQ.

Support for a positive attitude towards health should be included in al therapy programs. Moreover, trust in the health provider together with support from the provider should be simultaneously implemented because both factors depend on each other during the intervention. Therefore, the selection of a health provider with a high HQ, as well as the establishment of effective project management, will likely result in a successful comprehensive lifestyle modification in obesity (The Obesity Expert Panel, 2013). Furthermore, a high EQ among the providers also played an important role in client self-efficacy and self-care.

In practice, the results of this research may be applied to develop a practical, comprehensive strategy to combat obesity using a well-operated program. Health providers must have a high HQ and EQ, which can be measured and enhanced using the techniques of psycho-philosophy. Finally, a comprehensive program must consider the client’s attitude towards healthy, which represents the potential key to the success of the program. Moreover, trust in the health provider and support from the provider must also be ensured.

The recommendations for future research are as follows: 1) to study the relationship between socio-psychological factors and the client’s quality of life, and 2) to study or identify other socio-psychological variables that influence the client’s self-efficacy, self- care and self-regulation.

## 6. Conclusion

In obesity, the client’s attitude towards health is the only factor that significantly influenced self-efficacy, self-regulation and self-care (3SELF). At the health provider level, both the HQ and PM factors significantly influenced all of the 3SELF principles. Trust in the health provider, support from the provider, support from the team EQ and health knowledge significantly influenced only 1 or 2 of the 3SELF s. There was no cross-level interaction effect between some of the psychological and social factors of the health provider and the client that influenced the clients’ self-efficacy related to health care, self-regulation and self-care.
